# Recovery of phospho-ERK activity allows melanoma cells to escape from BRAF inhibitor therapy

**DOI:** 10.1038/sj.bjc.6605714

**Published:** 2010-06-08

**Authors:** K H T Paraiso, I V Fedorenko, L P Cantini, A C Munko, M Hall, V K Sondak, J L Messina, K T Flaherty, K S M Smalley

**Affiliations:** 1 Department of Molecular Oncology, The Moffitt Cancer Center and Research Institute, 12902 Magnolia Drive, Tampa, FL 33612, USA; 2 Department of Cutaneous Oncology, The Moffitt Cancer Center and Research Institute, 12902 Magnolia Drive, Tampa, FL 33612, USA; 3 Department of Pathology and Cell Biology, University of South Florida College of Medicine, 12901 Bruce B. Downs Boulevard, MDC 11, Tampa, FL 33612, USA; 4 Department of Medicine, Division of Hematology/Oncology, Massachusetts General Hospital Cancer Center, 55 Fruit Street, Yawkey 9E, Boston, MA 02114, USA; 5 Department of Integrated Mathematical Oncology, The Moffitt Cancer Center and Research Institute, 12902 Magnolia Drive, Tampa, FL 33612, USA

**Keywords:** melanoma, BRAF, resistance, therapy

## Abstract

**Background:**

Resistance to BRAF inhibitors is an emerging problem in the melanoma field. Strategies to prevent and overcome resistance are urgently required.

**Methods:**

The dynamics of cell signalling, BrdU incorporation and cell-cycle entry after BRAF inhibition was measured using flow cytometry and western blot. The ability of combined BRAF/MEK inhibition to prevent the emergence of resistance was demonstrated by apoptosis and colony formation assays and in 3D organotypic cell culture.

**Results:**

BRAF inhibition led to a rapid recovery of phospho-ERK (pERK) signalling. Although most of the cells remained growth arrested in the presence of drug, a minor population of cells retained their proliferative potential and escaped from BRAF inhibitor therapy. A function for the rebound pERK signalling in therapy escape was demonstrated by the ability of combined BRAF/MEK inhibition to enhance the levels of apoptosis and abrogate the onset of resistance.

**Conclusion:**

Combined BRAF/MEK inhibition may be one strategy to prevent the emergence of drug resistance in *BRAF*-V600E-mutated melanomas.

The discovery that ∼50% of human melanomas harbour activating V600E mutations in the serine/threonine kinase *BRAF* has raised the possibility that these tumours may be amenable to targeted therapy (Davies *et al*, 2002; Smalley and Flaherty, 2009). A large number of preclinical studies have now validated mutated *BRAF* as a *bona fide* therapeutic target in melanoma (Hingorani *et al*, 2003; Karasarides *et al*, 2004; Sharma *et al*, 2005). Mechanistically, mutated *BRAF* seems to exert most of its oncogenic effects through the activation of the RAF/MEK/ERK mitogen-activated protein kinase (MAPK) pathway (Karasarides *et al*, 2004; Wellbrock *et al*, 2004b). The MAPK activity drives the uncontrolled growth of melanoma cells by upregulating the expression of cyclin D1 and through the suppression of the cyclin-dependent kinase inhibitor p27^KIP1^ (Smalley, 2003; Bhatt *et al*, 2005).

A number of novel BRAF inhibitors have been described that are now at various stages of clinical development (King *et al*, 2006; Montagut *et al*, 2008; Tsai *et al*, 2008; Flaherty *et al*, 2009). Of these, PLX4032 and PLX4720 (Plexxikon/Roche, Nutley, NJ, USA), have been exciting great interest, with recent studies from our group and others showing these compounds to have excellent anti-tumour activity *in vitro* and *in vivo* (Cartlidge *et al*, 2008; Sala *et al*, 2008; Tsai *et al*, 2008). PLX4032 has been recently evaluated in a phase I clinical trial of melanoma patients harbouring the *BRAF*-V600E mutation (Flaherty *et al*, 2009). Responses were observed in an unprecedented 70% of patients, and there is now hope that small molecule BRAF inhibitors could constitute a major new melanoma therapy.

Although the clinical development of BRAF inhibitors is at an early stage, it is already clear that the impressive levels of response seen initially do not necessarily persist for extended periods of time. These observations mirror the pattern of response seen to targeted therapy in CML, GIST (Sawyers, 2004; Bauer *et al*, 2007) and most recently medulloblastoma (Rudin *et al*, 2009; Yauch *et al*, 2009), where an initial period of tumour regression is later followed by relapse. In this study, we have identified the rebound activation of phospho-ERK (pERK) as being a mechanism of early therapy escape and show that combined BRAF/MEK inhibition can both enhance the levels of apoptosis and abrogate the onset of resistance.

## Materials and methods

### Cell culture and growth inhibition

Melanoma cell lines were a gift from Dr Meenhard Herlyn (The Wistar Institute) and were genotyped as described in Haass *et al* (2008). Cells were plated into a 96-well plate at a density of 2.5 × 10^4^ cells per ml and left to grow overnight before being treated with increasing concentrations of PLX4720 in triplicate; after 72 h, the levels of growth inhibition were examined using the MTT assay (Smalley *et al*, 2007b). Data show the mean of at least three independent experiments±the s.e. mean. In all cases, ^*^ indicates statistical significance where *P*<0.05. PLX4720 was dissolved in 100% DMSO and stored at −20°C as a 10 mM solution. U0126 was from EMD Biosciences (Carlsbad, CA, USA) and was prepared in a similar manner to PLX4720.

### Western blotting

Proteins were extracted and blotted for as described in Smalley *et al* (2005). After analysis, western blots were stripped once and reprobed for *β*-actin or GAPDH to demonstrate even protein loading. The antibodies to pERK, cleaved caspase-3, phospho-RB protein, total-RB protein, PARP, CRAF, cyclin D1 and total-ERK were from Cell Signaling Technology (Beverly, MA, USA) and the antibody to p27 was from BD Biosciences (Franklin Lakes, NJ, USA).

### Flow cytometry

Cells were plated into 10-cm dishes at 60% confluency and left to grow overnight before being treated with PLX4720 (0.3 and 3 *μ*M) for 24 h. In other studies, cells were treated with PLX4720 (3 *μ*M) in the absence or presence of U0126 (3 *μ*M) and harvested after 24 or 48 h. Annexin-V labelling and propidium iodide staining were performed as described in Smalley *et al* (2007a).

### BrdU incorporation

Cells were seeded in 10 cm plates at a density of 100 000 cells ml^−1^ and grown overnight before being treated with PLX4720 (3 *μ*M) for 72 h or 1, 2, 3 and 4 weeks. For the 1-, 2-, 3- and 4-week treatments, PLX4720 (3 *μ*M) was added twice per week. One hour before the end of the drug treatment, BrdU (Sigma-Aldrich, St Louis, MO, USA) was added to the cells to a final concentration of 20 *μ*M for 1 h. Cells were fixed and permeabilised with eBioscience's fixation and permeabilisation buffers. The BrdU epitopes were exposed by incubating with DNase (Sigma-Aldrich) before staining with anti-BrdU conjugated to FITC (eBioscience, San Diego, CA, USA). In all, 7-AAD (BD Bioscience) was added to stain for DNA before acquisition on a BD Facscalibur flow cytometer.

### MEK1 sequencing

Sequencing of MEK1 Exons 3 and 6 was performed as described in Emery *et al* (2009).

### 3D spheroid assays

Melanoma spheroids were prepared using the liquid overlay method (Smalley *et al*, 2006). Spheroids were treated with 0.03–30 *μ*M of PLX4720 or U0126, PLX4720 (both 3 *μ*M) and both drugs in combination for 72 h before being washed (3 × in media) and treated with calcein-AM, ethidium bromide (Molecular Probes, Eugene, OR, USA) for 1 h at 37°C, according to the manufacturer's instructions. After this time, pictures of the invading spheroids were taken using a Nikon-300 inverted fluorescence microscope.

### Colony formation

Cells (1 × 10^4^ per ml) were seeded out into six-well plates and grown overnight before being treated with vehicle, PLX4720 (3 *μ*M), U0126 (3 *μ*M) or the two drugs in combination. Cells were left to grow for 4 weeks with new drug added twice per week. Media was aspirated, and plates were stained with crystal violet solution (50% methanol + 50% H_2_O + 0.5% crystal violet). Control plates were grown for 1 week in the absence of any drug, until 100% confluency was reached.

## Results

### PLX4720 has selective effects on *BRAF*-V600E-mutated melanoma cell lines

**Figure 1:**
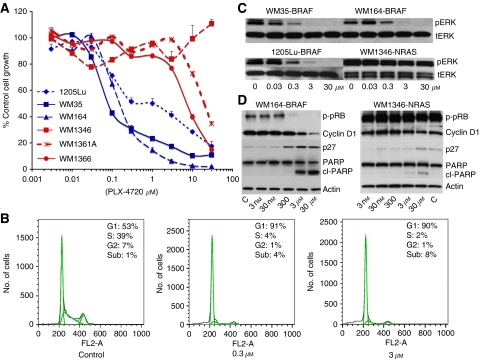
PLX4720 inhibits the growth of melanoma cells harbouring the *BRAF*-V600E mutation. (**A**). Increasing concentrations of PLX4720 reduced the growth of melanoma cell lines harbouring the *BRAF*-V600E mutation (WM35, 1205Lu and WM164), whereas melanoma cell lines that were *BRAF* wild type were relatively resistant (WM1346, WM1361A and WM1366). Cells were treated with drug (3 nM–30 *μ*M) for 72 h, and cell numbers were quantified using the MTT assay. Bars show s.e. mean. (**B**) Low doses of PLX4720 are cytostatic in melanoma cells harbouring the *BRAF*-V600E mutation. 1205Lu cells were treated were either 0.3 or 3 *μ*M PLX4720 for 24 h before being fixed, stained with propidium iodide and analysed by flow cytometry. (**C**) PLX4720 inhibits MAPK signalling in *BRAF*-V600E-mutated melanoma cells. Cells were treated with increasing concentrations of PLX4720 (0.03–30 *μ*M, 1 h); proteins were extracted and probed for expression of phospho-ERK (pERK). Blots were stripped once and reprobed for total-ERK to show even protein loading. (**D**) PLX4720 induces a concentration-dependent reduction in the phosphorylation of the retinoblastoma protein (phospho-RB), induces the cleavage of PARP, stabilises p27 and suppresses the expression of cyclin D1 in WM164 *BRAF*-V600E-mutated melanoma cells. Cells were treated with increasing concentrations of PLX4720 (3 nM–30 *μ*M) for 24 h, after which time, protein was extracted and resolved by western blotting (C=vehicle control). Blots were stripped once and probed for actin to show equal protein loading.

Treatment of melanoma cells with increasing concentrations of the BRAF inhibitor PLX4720 led to a dose-dependent reduction in the growth of *BRAF*-V600E-mutated melanoma cell lines (WM35, WM164 and 1205Lu) (Figure 1A). In contrast, cell lines that harboured an *NRAS* mutation (WM1346, WM1361A and WM1366) were more resistant (Figure 1A). Lower doses of PLX4720 (0.3 and 3 *μ*M) led to a profound G1-phase cell-cycle arrest and a reduction of 1205Lu cells entering into S-phase (Figure 1B). Increasing concentrations of PLX4720 (1 h) inhibited pERK signalling in three *BRAF*-mutated melanoma cell lines (WM35, WM164 and 1205Lu), but not an *NRAS*-mutated cell line (WM1346) (Figure 1C). It was noted that PLX4720 also reduced pRB protein phosphorylation, increased p27 expression, suppressed cyclin D1 expression and induced cleavage of PARP only in melanoma cell lines harbouring the *BRAF*-V600E mutation (Figure 1D).

### PLX4720-mediated apoptosis induction is *BRAF*-V600E-mutation specific

**Figure 2:**
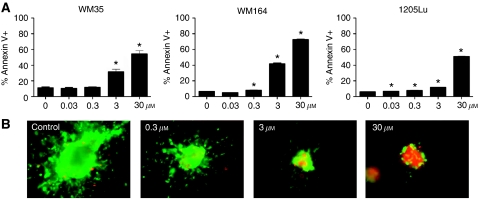
PLX4720 induces apoptosis in BRAF-V600E-mutated melanoma cell lines. (**A**) PLX4720 induces apoptosis in three *BRAF*-mutated melanoma cell lines. Cultures were treated with increasing concentrations of PLX4720 (0.03–30 *μ*M, 48 h), before staining for FITC-annexin-V and flow cytometry. Data show mean of three experiments. (**B**) PLX4720 reduces viability and invasion of 1205Lu cells grown as 3D collagen-implanted spheroids. Preformed 1205Lu spheroids were implanted into collagen and overlayed with media. Cells were treated with PLX4720 (0.3–30 *μ*M for 72 h) before being treated with calcein-AM and ethidium bromide. Green, viable cells; red, dead cells. Lack of green staining also indicates a loss of cell viability. Magnification × 10. ^*^P<0.05, Significant difference from control. The colour reproduction of this figure is available on the html full text version of the manuscript.

Concentrations of PLX4720 ⩾3 *μ*M were required for apoptosis induction across a panel of three *BRAF*-mutated melanoma cell lines (WM35, WM164 and 1205Lu) (Figure 2A). The pro-apoptotic effects of PLX4720 were found to be *BRAF* specific, with high levels (>30%) of apoptosis only induced in the *BRAF*-V600E-mutated melanoma cell line panel (WM35, WM164 and 1205Lu), and not the *NRAS*-mutated melanoma cell lines (WM1346, WM1361A and WM1366) (Supplementary Figure 1). The induction of apoptosis was found to be time dependent with apoptosis observed only >24 h. Often, the pharmacological profile of drugs in 2D culture is not predictive of response in 3D culture. Here, it was found that the concentrations of PLX4720 required (>3 *μ*M) to induce apoptosis in 2D cell culture (Figure 2B) were equivalent to those necessary for loss of spheroid viability (as shown by the reduction of green staining and increased red staining) (Figure 2B). Interestingly, some viable melanoma cells persisted even at the highest concentrations of drug.

### Some cells escape from PLX4720 treatment and become resistant

**Figure 3:**
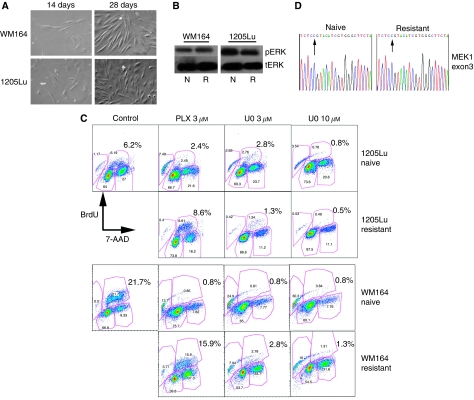
Melanoma cells escape PLX4720 and become resistant. (**A**) Representative photomicrograph of WM164 and 1205Lu melanoma cells treated with PLX4720 (3 *μ*M) for either 14 or 28 days. (**B**) Western blot showing levels of pERK expression in PLX4720 naive (N) and resistant (R) (8 weeks, 3 *μ*M) WM164 and 1205Lu cell lines. Note that the resistant cell lines were maintained continuously in the presence of PLX4720 (3 *μ*M). Total-ERK demonstrates even protein loading. (**C**) Resistant 1205Lu and WM164 cell lines continue to incorporate BrdU in the continual presence of PLX4720 (3 *μ*M). Panel shows either treatment-naive WM164 and 1205Lu cell lines (control) or resistant (chronically treated with PLX4720 for 8 weeks) treated with either PLX4720 (3 *μ*M) or the MEK inhibitor U0126 (3 and 10 *μ*M). Cells were stained for BrdU (20 *μ*M, 1 h) uptake and the cell viability marker 7-AAD and were analysed by flow cytometry. (**D**) Representative sequencing trace from Exon 3 of MEK1 of 1205Lu cells chronically treated with PLX4720 for 8 weeks, arrow indicates site of P124L mutation identified previously in Emery *et al* (2009).

We next asked whether *BRAF*-V600E-mutated melanoma cells escaped from PLX4720 therapy and become drug resistant. Here, melanoma cell lines (WM164 and 1205Lu) were treated with PLX4720 (either 2 or 3 *μ*M) over a 2-month period with fresh drug added twice per week. It was noted that after an initial round of cell death, a limited number of viable cells remained and as treatment progressed, these clones began to regrow (>28 days) and eventually repopulated the whole culture (Figure 3A). The drug-resistant phenotype of the surviving cells was demonstrated by the ability of both cell lines to maintain their pERK signalling and incorporate BrdU in the continuous presence of PLX4720 (3 *μ*M) (Figure 3B and C). In contrast, PLX4720 treatment (3 *μ*M) potently inhibited BrdU incorporation in the PLX4720-naive WM164 and 1205Lu cell lines (Figure 3C). It was further shown that the proliferation of the PLX4720-resistant WM164 and 1205Lu cell lines was dependent on MAPK signalling, with MEK inhibitor treatment (U0126; 3 and 10 *μ*M) preventing the incorporation of BrdU (Figure 3C).

Earlier studies have suggested that acquired resistance to the MEK inhibitor AZD6244 occurs as the result of an acquired mutation in MEK1 (Emery *et al*, 2009). As BRAF and MEK lie in the same signal transduction pathway, we sequenced Exons 3 and 6 of MEK1 for both WM164 and 1205Lu cell lines. It was found that neither of the PLX4720-resistant cell lines acquired the P124L or Q56P mutations in MEK1 (Figure 3D and data not shown).

### Prolonged PLX4720 treatment leads to a recovery of pERK signalling

**Figure 4:**
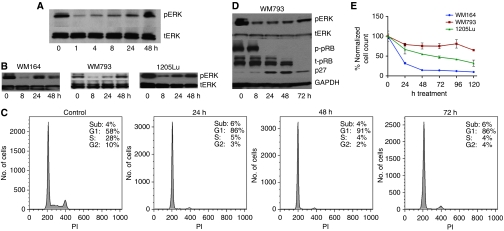
pERK signalling recovers after PLX4720 treatment. (**A**) Naive WM164 melanoma cells were treated with PLX4720 (3 *μ*M, every 24 h) for increasing periods of time (0–48 h) and probed for pERK and total-ERK (tERK). (**B**) Recovery of pERK is observed in three naive *BRAF*-V600E-mutated melanoma cell lines. Cells were treated with PLX4720 for 0, 8, 24, 48 h (3 *μ*M) and analysed as in (A). (**C**) Most cells remain growth arrested even when pERK recovers. WM793 cells were treated with PLX4720 (3 *μ*M) for 0–72 h. Cells were harvested, fixed and stained with propidium iodide before being analysed by flow cytometry. (**D**) p27 expression levels remain high even when pERK signalling recovers. WM793 cells were treated with PLX4720 as for (**C**); protein lysates were probed for expression of pERK, total-ERK (tERK), phospho-RB (p-pRB), total retinoblastoma protein (t-RB) and p27. Equal protein loading was confirmed by stripping the blot once and probing for GAPDH expression. (**E**) PLX4720 treatment leads to a drop in cell numbers followed by stabilisation of the population. WM793, 1205Lu and WM164 melanoma cells were treated with PLX4720 (3 *μ*M) for 0–120 h. At each time point, the cells were removed from the plate and counted. Data show the mean±s.e.mean of three independent experiments.

Having shown the reliance of the PLX4720-resistant melanoma cell lines on MAPK signalling, we next investigated the time course of pERK signalling recovery. Treatment of drug-naive WM164 cells with PLX4720 (3 *μ*M) showed the pathway to be rapidly inhibited, with some recovery of signalling >24 h (Figure 4A and B). The recovery of pERK signalling observed was found to be insensitive to repeated PLX4720 treatments (drug added every 24 h) (Figure 4A). To explain the apparent anomaly between the recovery of pERK signalling >24 h and the profound growth arrest/apoptosis observed at 48 and 72 h (Figures 1A and 2A), we next investigated the cell cycle and signalling profile of cells treated with PLX4720 over a 72-h period. These studies showed that even though pERK signalling recovered, the majority of the cells remained growth arrested (Figure 4C), and that this was associated with increased p27 expression and hypophosphorylation of the pRB protein (Figure 4D). Interestingly, a minor population of cells were identified that continued to proceed through S-phase (Figure 4C). The existence of a minor proliferating subpopulation was also confirmed by BrdU incorporation assays (1–4 weeks), with studies showing that 2–4% of WM164 and WM793 cells continued to incorporate BrdU in the continuous presence of PLX4720 (3 *μ*M) (Supplementary Figure 2).

Cell counting experiments were performed to better understand how PLX4720-induced apoptosis, cell-cycle arrest and therapy escape impacted on the population as a whole (Figure 4E). It was observed that after an initial drop in cell numbers, the population remained relatively stable, suggesting that the recovery of pERK signalling attenuated the anti-melanoma activity of PLX4720.

### Rebound pERK treatment allows for escape from PLX4720-mediated apoptosis

**Figure 5:**
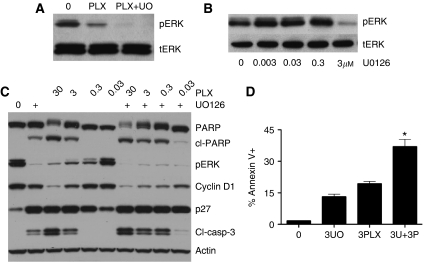
The function of rebound pERK signalling in the escape from PLX4720 treatment. (**A**) U0126 blocks the rebound increase in pERK after PLX4720 treatment. Melanoma cells were either treated with vehicle (0), PLX4720 (3 *μ*M) or PLX4720 + U0126 (both 3 *μ*M) for 48 h, protein was then probed for expression of pERK and tERK. (**B**) Melanoma cells were treated with increasing concentrations of U0126 for 1 h before being probed for pERK and tERK expression. (**C**) Cells were treated with increasing concentrations of PLX4720 (30 nM–30 *μ*M) for 24 h in the absence or presence of U0126 (3 *μ*M), after which time, protein was extracted and resolved by western blotting and probed for either cleaved PARP (cl-PARP), phospho-ERK (pERK), cyclin D1 (Cyclin D1), p27 or cleaved caspase-3 (cl-casp-3). Blots were stripped once and probed for actin to show equal protein loading. (**D**) Combined BRAF and MEK inhibition leads to enhanced apoptosis. WM164 cells were treated with either vehicle, U0126 (3 *μ*M, 3U0), PLX4720 (3PLX, 3 *μ*M) or the two inhibitors in combination for 48 h. Levels of apoptosis were measured by annexin-V staining and flow cytometry. Data show the mean of three experiments. ^*^P<0.05.

Having demonstrated that pERK signalling recovered after PLX4720 treatment, we next determined whether dual BRAF/MEK inhibition led to enhanced cytotoxicity. It was noted that although the recovery of pERK signalling was insensitive to repeated PLX4720 treatments (Figure 4A), rebound pERK signalling was sensitive to the MEK inhibitor U0126 (3 *μ*M) (Figure 5A and B). Combined treatment of drug-naive WM164 cells with both PLX4720 and U0126 was found to decrease the expression of cyclin D1 (Figure 5C) and enhance the level of PLX4720-induced PARP and caspase-3 cleavage (Figure 5C). In contrast, expression of p27, a protein relatively sensitive to BRAF/MEK inhibition, was little enhanced when PLX4720 and U0126 were combined. The western blotting results were also mirrored in apoptosis assays, with the addition of U0126 (3 *μ*M) significantly enhancing the pro-apoptotic activity of low-dose PLX4720 (3 *μ*M) in drug-naive WM164 cells at both 24 and 48 h (Figure 5D).

### Combined BRAF/MEK inhibitor treatment prevents the acquisition of resistance

**Figure 6:**
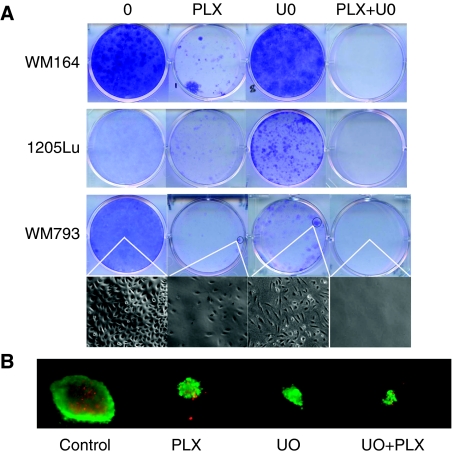
Dual BRAF/MEK inhibition prevents escape from PLX4720 therapy. (**A**) WM164, WM793 and 1205Lu melanoma cells were treated with vehicle (1 week), PLX4720 (3 *μ*M), U0126 (3 *μ*M) or the two inhibitors in combination (both 3 *μ*M) for 4 weeks. After this time, colonies were fixed and stained with crystal violet. Photographs are representative of three independent experiments. Photomicrographs show the detail of one colony each on the WM793 plate ( × 4). (**B**) Combined PLX4720 and U0126 treatment reduce growth of melanoma cells and enhance cell death in a 3D spheroid model. WM164 spheroids were implanted in collagen and treated with PLX4720 (3 *μ*M), U0126 (3 *μ*M) or the two drugs in combination for 72 h. After this time, plates were washed and cells were stained with a cell viability kit. Red=dead cells, green=live cells. The colour reproduction of this figure is available on the html full text version of the manuscript.

In a final series of experiments, we explored whether dual BRAF/MEK inhibition blocked the MAPK-dependent escape from PLX4720 therapy and asked whether this prevented the onset of resistance. Here, WM164, WM793 and 1205Lu cells were treated with PLX4720 (3 *μ*M), U0126 (3 *μ*M) or the two inhibitors in combination for 4 weeks. It was noted that although PLX4720 was more effective at reducing colony formation than U0126 (Figure 6A), a number of clones did remain. In contrast, treatment with U0126 and PLX4720 in combination completely inhibited the formation of all colonies. A thorough microscopic examination of the plates revealed that no cells remained (Figure 6A, see inset). Examination of the vehicle control plates showed the cells to be highly confluent. It was further found that the combination of PLX4720 and U0126 (both 3 *μ*M) also reduced the growth and survival of melanoma cell lines grown as 3D collagen-implanted spheroid cultures (Figure 6B).

## Discussion

The past 30 years have seen little improvement in the treatment of disseminated melanoma. After the recent success of targeted therapy agents such as imatinib mesylate in chronic myeloid leukemia, there is now hope that melanoma may be amenable to similar strategies. A recent phase I clinical trial of the BRAF inhibitor PLX4032 has validated this concept and showed that most patients whose melanomas harboured the *BRAF* V600E respond well to this treatment (Flaherty *et al*, 2009). Although long-term follow-up data are not currently available, early indications suggest that most PLX4032-treated patients eventually become resistant. In this study, we have focused on the earliest stages of therapy escape after treatment with the BRAF inhibitor PLX4720. Through an initial series of experiments, we confirmed that PLX4720 had good selectivity for *BRAF*-mutated melanoma cell lines over those harbouring *NRAS* mutations and also demonstrated that PLX4720 was able to induce significant levels of apoptosis. The induction of apoptosis induced was slow in onset (>24 h), but very *BRAF* specific, with very little apoptosis observed in melanoma cell lines that were *BRAF* wild type.

Currently, very little is known about the mechanism of early therapy escape after BRAF inhibition. In non-melanoma systems, chronic treatment with the MEK inhibitor CI-1040 leads to resistance associated with increased KRAS and MEK expression (Wang *et al*, 2005). In melanoma, it has been shown that both growth factors and cytokines rescue cells from apoptosis after siRNA-induced knockdown of BRAF (Christensen and Guldberg, 2005; Gray-Schopfer *et al*, 2007). Recent work has also suggested that resistance of melanoma patients to the MEK inhibitor AZD6244 is associated with mutations in MEK1 (Emery *et al*, 2009). Other studies have shown that acquired BRAF inhibitor resistance after long-term drug treatment is associated with pathway switching, where MAPK signalling is routed from BRAF to CRAF (Montagut *et al*, 2008).

This study makes the unexpected observation that combined BRAF and MEK inhibitor treatment enhances the levels of apoptosis before resistance to BRAF inhibition is even acquired, suggesting that the recovery of melanoma signalling occurs much earlier than previously suspected. The observation that dual MEK/BRAF inhibition blocks colony formation also argues that rebound MAPK signalling observed has a key function in the escape from therapy. Although targeting the same pathway at two points seems redundant, it is likely that dual inhibition may be a good strategy to counteract the feedback inhibition loops that are relieved after pathway blockade at a single point (Pratilas *et al*, 2009). Intriguingly, the possibility also exists that MEK and BRAF inhibitors may hit subtly different cellular targets. There is already evidence that both ARAF and CRAF affect pathways other than MEK, and although not well characterised, it is possible that other BRAF targets may exist (Wellbrock *et al*, 2004a).

The finding that dual BRAF/MEK inhibition prevents the onset of resistance in our *in vitro* melanoma models suggests that MEK inhibitors may be of use in managing resistance to BRAF inhibitors and may delay or even prevent the onset of resistance in some cases. These findings provide a strong rationale for the testing of combined BRAF and MEK inhibitors in the clinical setting.
