# Supplementary material for: Recovery of phospho-ERK activity allows melanoma cells to escape from BRAF inhibitor therapy
Source: Br J Cancer. 2010 Jun 8;102(12):1724–30. doi: 10.1038/sj.bjc.6605714 (PMC2883709; doi:10.1038/sj.bjc.6605714)
Supplement: Supplementary Figures [file 6605714x1.doc]

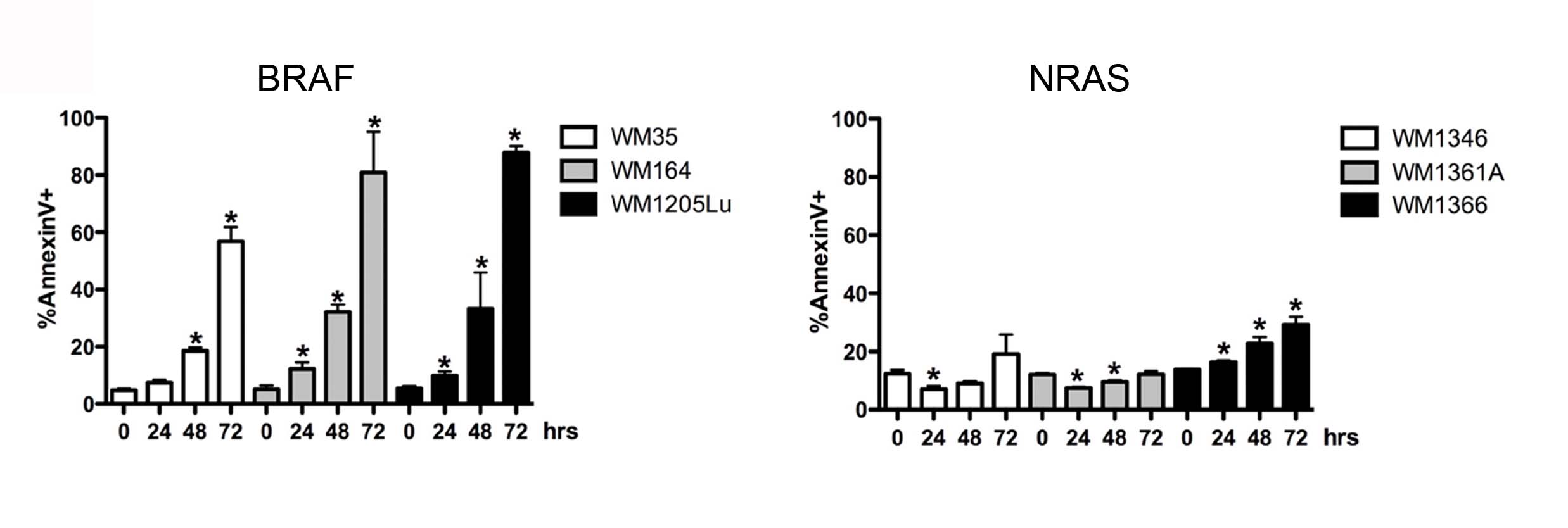


**Supplemental Figure 1:**

PLX4720 selectively induces apoptosis in melanoma cell lines harboring a *BRAF* V600E mutation but not those harboring an *NRAS* mutation. Melanoma cell lines were treated with PLX4720 (30 μM) for increasing periods of time (24-72 hrs), before being stained with FITC-annexin-V.

**
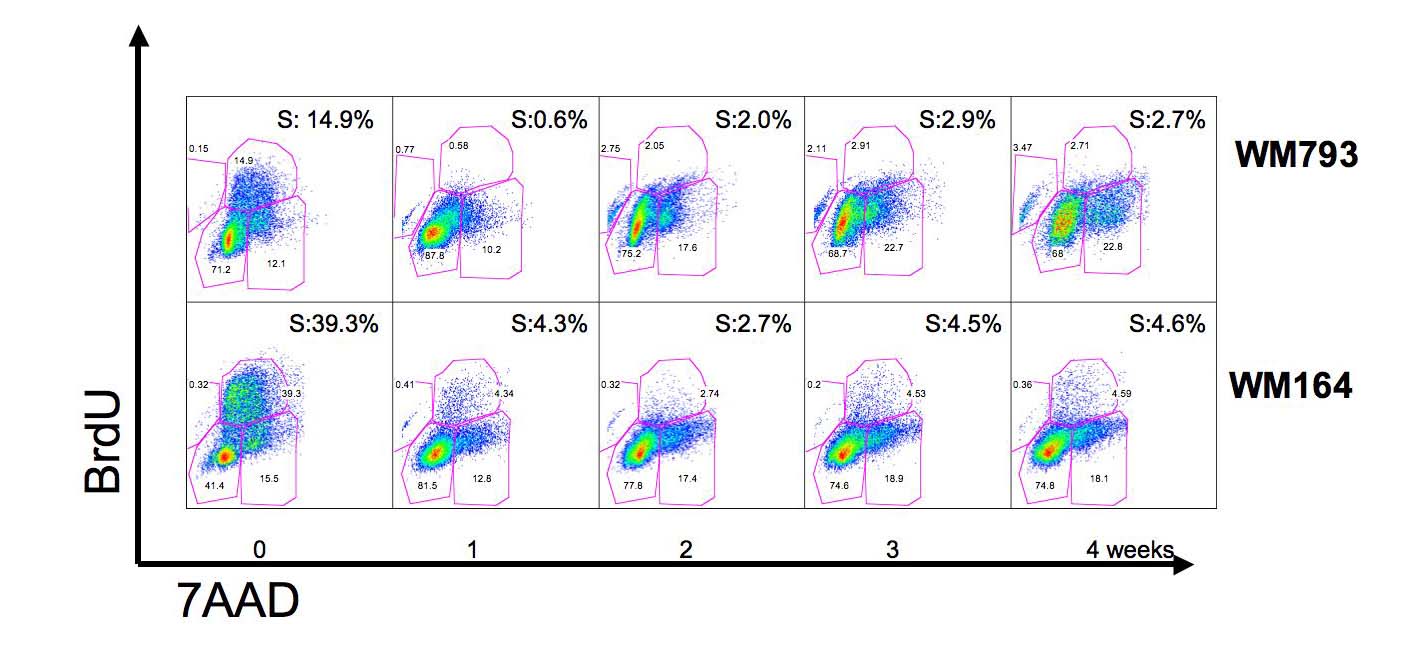
**

**Supplemental Figure 2:**

A minor population of cells continues to incorporate BrdU in the continual presence of PLX4720 (3 μM). WM164 and 1205Lu cells were treated with PLX4720 over 1-4 weeks. At each time point, cells were pulsed with BrdU for 1 hr and then stained for anti-BrdU and 17-ADD. Numbers indicate the percentage of cells in S-phase.
